# Not Just “COVID Anxiety”: A Case of Subacute Thyroiditis in a Patient Recovering From SARS-CoV-2 Infection

**DOI:** 10.7759/cureus.24236

**Published:** 2022-04-18

**Authors:** Hameed Ullah, Ijaz Ali, Fazal Alam, Wajid Ali, Masroor Anwar

**Affiliations:** 1 Internal Medicine, Hayatabad Medical Complex Peshawar, Peshawar, PAK; 2 Internal Medicine, Khyber Teaching Hospital, Peshawar, PAK

**Keywords:** painful thyroiditis, tfts, covid anxiety, anterior neck pain, covid-19, hyperthyroidism, subacute thyroiditis

## Abstract

Subacute thyroiditis (SAT) is an uncommon, granulomatous, inflammatory thyroid disorder. It usually presents with anterior neck and/or jaw pain, diffusely tender goiter, fever, fatigue, myalgia, and anorexia. Most patients with SAT initially develop symptoms and signs of hyperthyroidism which usually subsides within a few weeks with or without going through a transient phase of hypothyroidism. SAT is usually associated with a viral infection of the upper respiratory tract. We report a case of SAT in a 30-year-old male with a recent COVID-19 infection. The patient presented with a three days history of painful anterior neck mass and palpitations. He was diagnosed with COVID-19 16 days before presentation. His infection was mild and did not need any treatment apart from as-needed paracetamol. The patient was found to have a clinical, laboratory, and imaging findings consistent with SAT. The patient was prescribed ibuprofen, prednisone, and propranolol. The patient showed significant clinical and biochemical improvement on follow-up visits, achieving a euthyroid state within several weeks. Like many other respiratory viral illnesses, COVID-19 also seems to be associated with SAT. Other endocrinological sequelae have also been reported. While reviewing patients suffering from COVID-19 infection, these possibilities should be kept in mind.

## Introduction

Subacute thyroiditis (SAT) is an uncommon cause of hyperthyroidism [1]. The most common presenting symptom is anterior neck pain [2]. Other common symptoms include fever, fatigue, anorexia, myalgia, and malaise [1]. The thyroid gland is diffusely or asymmetrically swollen and tender. Almost 60% of the patients develop symptoms and signs of hyperthyroidism [3]. The disease is usually self-limited and follows a predictable course of hyperthyroidism followed by a transient phase of hypothyroidism. Most patients achieve a euthyroid state within two to four months; however, a subset of the patients can develop a chronic state of hypothyroidism [2]. We report a case of SAT in a 30-year-old male who was recovering from a mild SARS-CoV-2 infection.

## Case presentation

A previously healthy, 30-year-old male presented to the ED with a three days history of pain in the anterior neck and chin, palpitations, and anxiety. The patient revealed that 16 days ago, he was diagnosed with COVID-19 infection following his visit to the hospital due to complaints of fever and generalized body aches. His infection was mild and did not need any treatment apart from as-needed paracetamol. He was making an uneventful recovery until three days ago, when he developed these symptoms. He went to a nearby clinic, where he was reassured that “COVID anxiety” was the cause of his symptoms.

**Figure 1:**
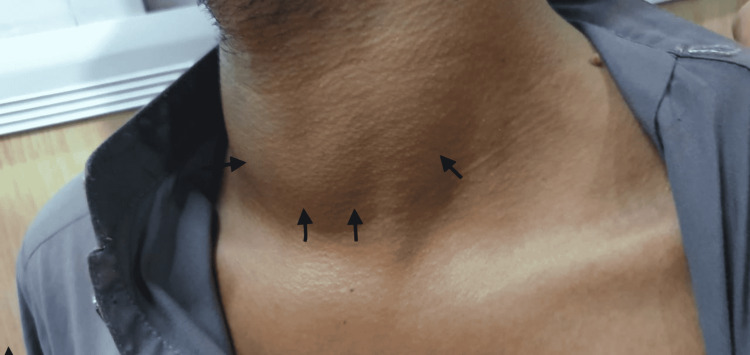
The swollen thyroid gland on initial presentation.

On examination, the patient was found to have tachycardia (heart rate = 134 bpm), a blood pressure of 131/73 mm Hg, oxygen saturation of 98% on room air, and a random blood glucose level of 97 mg/dL. He had warm, sweaty palms and an exquisitely tender neck mass consistent with a diffusely swollen thyroid gland (Figure 1). No lymphadenopathy was observed. The rest of the examination was unremarkable. Laboratory investigations showed severely decreased serum thyroid-stimulating hormone (TSH) levels and highly elevated levels of serum T3 and T4. ECG showed sinus tachycardia. Along with lymphocytic leucocytosis, the elevation of erythrocyte sedimentation rate (ESR) and C-reactive protein (CRP) was also noted. Key laboratory values are summarised in Table 1. Ultrasonography of the neck showed diffuse enlargement, hypoechogenicity, and decreased vascularity of the thyroid gland on color Doppler. Due to high clinical suspicion of SAT, a radionuclide thyroid scan was deferred.

**Table 1:** Key laboratory values on initial presentation. ESR: Erythrocyte sedimentation rate; CRP: C-reactive protein; ALT: Alanine transaminase; TSH: Thyroid-stimulating hormone.

Test	Results	Normal values
Hemoglobin	12.97	11.5-17.5 g/dL
WBC	19.5 K	4-11 K/µL
Neutrophils	11.61%	40-75%
Lymphocytes	83.09%	20-45%
Platelets	497	150-450 k/µL
ESR	88	0-20 mm/ 1st hr
CRP	12	<0.5 mg/dL
Creatinine	0.7	0.64-1.2 mg/dL
Urea	22	18-45 mg/dL
ALT	73	10-50 U/L
Alkaline Phosphatase	104	40-129 U/L
TSH	0.005	0.3-4.2 mIU/L
T3	5.13	0.8-2.0 ng/mL
T4	24.86	5.1-14.1 µg/dL

**Figure 2:**
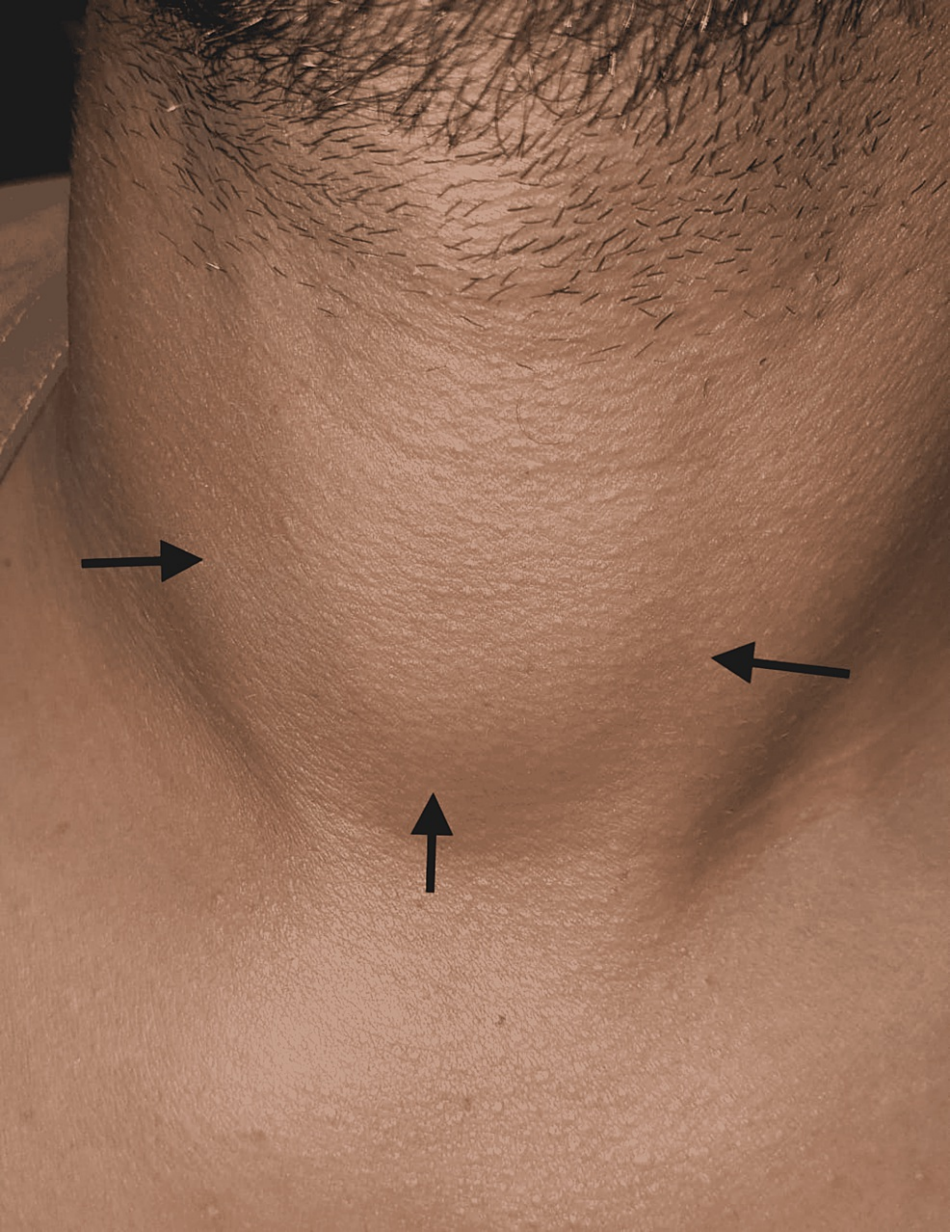
Complete resolution of the thyroid swelling.

The patient was reassured and discharged home on ibuprofen (400 mg three times a day), prednisone (30 mg daily), and propranolol (40 mg two times a day). On subsequent reviews, the patient's symptoms, signs, and thyroid function tests (TFTs) gradually improved. The patient was taken off the ibuprofen, and the dose of prednisone was gradually tapered over the next seven weeks. Seven weeks after the initial presentation, the patient's TFTs returned to normal and the thyroid swelling completely resolved (Figure 2). Monthly follow-up visits were scheduled for the possible development of hypothyroidism, but the patient's TFTs remained stable over the next four months.

## Discussion

SAT is the most frequent cause of painful thyroiditis [2]. Data suggests that it is caused by viral infections or postviral inflammatory responses. Many patients have a recent history of upper respiratory tract infection. Many viruses, including coxsackievirus, measles, and mumps, have been implicated in the development of SAT [4]. Since the beginning of the COVID-19 pandemic more than two years ago, many extrapulmonary manifestations of the disease, including endocrinological manifestations have been recognized. The first case of SAT secondary to COVID-19 was reported by Brancatella A et al. in early 2020 [5]. Ever since then, multiple cases of COVID-19 associated SAT have been reported from around the world. Most of these cases have been reported to occur either during the active infection or within a few weeks after the recovery. We add to the existing literature by reporting a case from Pakistan, with emphasis on the fact that symptoms and signs of hyperthyroidism in these patients can easily be mistaken as “COVID anxiety”. Severe anxiety and other psychiatric disorders have been reported in patients recovering from COVID-19 [6]. Since anxiety and hyperthyroidism share many clinical features, the diagnosis of SAT can easily be missed in these patients. Our patient was first discharged home with the reassurance that the symptoms were due to “COVID anxiety”, and will subside soon.

For COVID-19 to invade human cells, the presence of an angiotensin-converting enzyme 2 (ACE2) receptor is essential. The thyroid gland has been shown to express a significant amount of ACE2 receptors [7]. However, there is no evidence to suggest that direct viral invasion of the gland is the cause of SAT. Instead, a more likely explanation is that the inflammatory reaction to viral infections results in the activation of cellular host responses that then damage thyroid follicular cells [8].

Management of SAT includes alleviation of pain and inflammation with non-steroidal anti-inflammatory drugs (NSAIDs) and steroids [9, 10]. Patients with symptomatic hyperthyroidism also need treatment with a beta-blocker such as propranolol or atenolol.

## Conclusions

While reviewing patients with COVID-19 and anxiety, we must be mindful of the possibility of hyperthyroidism secondary to SAT, as it is easy to confuse symptoms of hyperthyroidism with anxiety. As the diagnosis of SAT is primarily clinical and timely recognition and prompt treatment can help alleviate most of the patient’s symptoms, a thorough history and physical examination are of paramount importance. 

## Appendices

Patient’s Perspective

I thought I was dying of this new virus. It was as if my heart wanted to come out of my chest. After the medications, I started to feel much better. I am thankful to God and the doctors. 
